# The first president's pathway into ESTSS: memories and ideas for future issue. Patients as partners

**DOI:** 10.3402/ejpt.v4i0.21308

**Published:** 2013-06-06

**Authors:** Erica M. Van der Schrieck-De Loos

**Affiliations:** Quality and Patient Safety in Healthcare, CBO Dutch Institute for Healthcare Improvement (a TNO company), Utrecht, The Netherlands

**Keywords:** Traumatic stress care team, psychotraumatology, patient involvement, quality and patient safety in healthcare, ESTSS main objectives

## Abstract

This article addresses Wolter De Loos’ pathway into ESTSS that ended on 6th January 2004. His footsteps are still printed in the field of psychotraumatology as he showed the field how a physician was trying to integrate his working field with that of psychiatry, with a great passion for his patients. Erica M. Van der Schrieck-De Loos outlines the perspectives of her father as funding president of the ESTSS by using his Opening Address of the Fourth ESTSS Conference in 1995. This address emphasized that the mixture of righteousness and offence in warfare is ubiquitious and everlasting. The perspective of the first president's pathway has been integrated with the current vision of the author to show that the patient can be a partner of the healthcare team. A suggestion for ESTSS main objective number (8), contained within the ESTSS mission statement, is to involve patients as partners of the traumatic stress care team into accelerating traumatic stress care across Europe and beyond.

ESTSS invited me to contribute to the 20th birthday. As the daughter of Wolter De Loos, I would like to present some of my father's ideas and reflections in relation to psychotraumatology as well as integrate thoughts from my own perspective into today's challenges for ESTSS. In ESTSS’ opinion Wolter had a tremendous input on the European psychotraumatology having been the first president and by forming ESTSS together with the other pioneers in the field. He was given the Honorary life membership of ESTSS and he is “Supervisor” of the highest honor given by ESTSS: the Dr. De Loos award for Distinguished Contribution to Psychotraumatology in Europe. Wolter De Loos, MD, PhD died in January 2004. He dedicated his life to psychotraumatology, patient's care, and ESTSS.

## Biography and pathway into ESTSS

After his residency in internal medicine (1980–1984), and his PhD in 1988, Wolter De Loos worked in the field of psychotraumatology for most of his life, 10 years in the University Hospital in Leiden (1983–1993), and 10 years in Utrecht, at the University Medical Center and Central Military Hospital (1993–2003). At the same time, he was working as a consultant for an eye clinic in the same university hospital. Wolter kept a dual interest throughout his life:

I favoured endocrinology but I also enjoyed intensive care medicine as a challenge in basic pathophysiological thinking and reasoning. (…) (Vermetten, 2004)

And with this last sentence written by Wolter's hand in 2003, his pathway into ESTSS has ended in some way, although Wolter's footsteps are still printed in the field of psychotraumatology as he showed the field how a physician was trying to integrate his working field with that of psychiatry, with a great passion for his patients. In this article, the first president's pathway continues by showing how to involve the patient during his or her care pathway. I would like to start with sharing some parts of the Opening Address by the First President of the European Society for Traumatic Stress Studies at the Fourth European Conference on Traumatic Stress in Paris, 7–11 May 1995.

## Opening Address at the Fourth European Conference on Traumatic Stress, Paris 1995 by Wolter De Loos

First of all, I want to welcome all of you together who have come to Paris as participants of the Fourth European Conference on Traumatic Stress. The purpose of these European Conferences is to develop the professional field of psychotraumatology. (…)

I have a special reason to tell you how the ESTSS has come to the idea of asking Louis Crocq to take this up. In 1988, I visited a psychosomatic conference in Marburg, Germany, which was organized by professor Wolfram Schüffel. It was he who had taken up responsibility for the care of victims of the mining disaster in Borken. Wolfram Schüffel took the initiative to organize a European Concerted Action for the Study of Disaster. In this group, thanks to Wolfram Schüffel, I first met Louis Crocq. (…)

I want to explain to you why it is so important that this German, Wolfram Schüffel, was pivotal in bringing together these Europeans in the field of psychotraumatology. On this historical day, this is of special value. It seems to me that the Germans, as a whole, still have feelings of uncertainty concerning World War II. For many of them, the War we commemorate today is still connected with embarrassing and disturbing feelings. The other nations cannot fully solve this problem for them. I personally believe that it is possible and that it would be important for them to take up the same tasks and engage in the same business as the nations around them. I believe that this may be a genuine way for the Germans to free themselves from inhibiting feelings of shame, guilt, and loss of honor. Of course, the fear for vicarious traumatization also remains within them. In this respect, one should realize that the present generation of professionals have, for the greater part, not been traumatized directly during the war and that it is a problem that also occurs in surrounding countries.

Psychotraumatology as a working field or a thematic domain in health care is not independent from the political situation and the historical development of a country and a continent. This is all too obvious for Europe, now and during the past 50 years. It has taken so much time to realize that there are no limits to what people can inflict on each other and that there are no people one can assume to be naturally immune to commit violence themselves. As much as there are no innocent people, there are no innocent countries. Since the end of World War II, many governments and individuals have fought many other wars. It is not possible to fight a war while keeping your hands clean. Coming back to the individual level that we know, now perhaps better than before, in every victim one may also find a perpetrator and vice versa. We know that many perpetrators have been victims earlier in their lives, for instance in cases of child abuse and incest. (…)

The mixture of righteousness and offense in warfare is ubiquitous and everlasting. A Dutch survivor of the concentration camp of Dachau, who was interviewed by the Süddeutsche Zeitung at the occasion of the 50th commemoration of his liberation by the 42nd “Rainbow” Infantry Division of the U.S. Army, told this story:

The American soldiers went into the barracks and came out again, white as chalk and vomiting. They could not understand how we could be so happy. Then I saw how 5 or 6 young SS men came down from a watch tower. I spoke to one of them. He had been conscripted at his age of 17 only a few weeks before; he showed me his pay book. While I was speaking to him, an American soldier shot him down, his blood splashing on me. I was outraged. I yelled out to the American ‘How dare you, you fucking son of a bitch’. I thought of the mother of this boy: ‘What had he done?’. A fortnight in uniform and been sent out to that watch tower.

When we were in Dachau for the actual commemoration last week, this survivor also commented to me:

Again, I saw happening what we had tried to resist during this war.

This is the problem of the victim, the victor, and the perpetrator.

The end of World War II was characterized by intense denial of its potential psychological aftermath. A small number of pioneers recognized the typical psychological effects of the tremendous traumatization in these Nazi extermination and concentration camps, forced labour, underground resistance, years of hiding, and so on. The impact of the Japanese camps was recognized even later. Not only prisoners of war, but also the entire Dutch population of Indonesia had been kept prisoner in concentration camps. The men between 18 and 60 years were forced to labour, many of them on the Birma railway, and in many cases women and children were also forced to work or to serve their victors sexually.

One of the important lessons of the massive traumatization of extensive groups of the European wartime population has been how long it took before the problems became evident in their full scope. Many survivors managed to live with subclinical disturbances for decades of up to 40 years or more. It has been striking how long people resisted confessing that they were in terrible distress. (…)

Why is it so important to repeat all of this at the opening of a scientific and professional conference? Well, it is the value of these experiences when it comes to predict our expectations for so many other clusters of massive traumatization. It literally takes a lifetime to make up the balance and I would not call anybody lucky before his death. (…)

There is more than the problem of the time lag of clinical presentation and recognition of a post-traumatic syndrome. (…)

Nowadays, we have realized that a number of other well-described psychiatric disorders should be seen as post-traumatic syndromes. According to ontogenic development, they may be listed as:

1. borderline personality disorder,
2. dissociative disorder,
3. somatoform and functional disorder,
4. post-traumatic stress disorder, and
5. post-traumatic personality disorder.

Looking back, again, to what the aftermath of World War II has taught us, we have seen that the way traumatized individuals signaled their distress, was more often at a somatic level or at the level of employment problems, social isolation, moving house frequently, emigration, and so on; more often, probably, then seeking help for their distressing memories, nightmares, feelings of guilt, and other direct psychological symptoms. In the Nordic, German, French, and Dutch literature of the first two decades after the War, the concept of premature senescence, in German “vorzeitige Alterung, Vergreisung oder Seneszenz”, in French “la sénescence prématurée”, was a commonly used entity. There was also circumstantial interest in late somatic effects, in German “Spätschäden”, according to the general psychosomatic theories of that time.

From a biological point of view, there are good reasons why physical symptoms are so often the first signals of post-traumatic syndromes. The core of the response to any form of existential menace is the inborn repertoire for survival behavior present in all mammals. In its essence, it has been described as the “defence reaction” (DR) by Walter Cannon in the first decades of this century. The physiology and functional anatomy of the DR have been studied in more detail and have been discriminated from the “general adaption syndrome” (GAS), the other well-known stress response described by Hans Selye in the 1930s. From a psychodynamic point of view, this is related to unsolvable conflict. The DR is characterized by arousal, the GAS by inhibition.

When considering the psychophysiology of the DR, the symptoms of many functional syndromes can be well understood as forms of recurrent or persistent alarm. Well-known examples include: hyperventilation, hyperdynamic β-adrenergic circulation, adrenal exhaustion, hyperkinetic heart syndrome, effort syndrome, autonomic dysfunction syndrome, neurasthenia, epidemic neuromyasthenia, benign myalgic encephalomyelitis, irritable bowel syndrome, allergic to everything, sugar intolerance, reactive hypoglycemia, and a number of more modern connotations. (…)

With regard to the diagnostic symptoms, we usually employ, these considerations cause some difficulties in integrating them into the existing classifications. In fact, the somato-psychic or psycho-somatic interactions have always been a twilight zone in our understanding of human functioning. The Diagnostic and Statistical Manual of Mental Disorders (DSM) has tried to shovel them together, but without true integration. Also, the psycho-somatic classifications I started to use about 10 years ago lack this integration. I see only one way to attain real integration and that is by constructing a matrix of dimensions. All observable aspects of an individual's functioning should then be broken down into these dimensions:

1. biological or physiological phenomena and inborn, autonomic responses;
2. behavioral programs, both motoric and associative;
3. semantic or declarative levels; and
4. relational behavior.

The matrix of these diagnostic dimensions is a construct for the full integrity of the individual patients. It corresponds to the meaning of a matrix in mathematics or electronics.

In the case of the somatoform disorders, these dimensional diagnostics would yield the following results. Psychophysiology is at first level the biological dimension corresponding to the phylogenic development of all species. Interpretation of a symptom can be an associative process. Conversion is avoidance to association with certain memories. Associations are automized but acquired basic programmes. This dimension is acquired by the individual during ontogeny as the first layer over its inborn properties. In a phylogenic respect, this can be shared by different species, animals, and humans. This is different for the third level, the semantic or declarative one. Humans learn to declare their emotions and they start to learn from their own psychophysiological sensations, which become incorporated in associative programmes. Incomplete emotional declaration is known as alexithymia and is a basic phenomenon in somatoform and functional disorders. Finally, the fourth level is the relational and this is an important feature of most somatoform disorders.

I have tried to draw you a picture of how a physician has tried to integrate his working field with that of psychiatry. I shall conclude my address by showing you a few pictures drawn by a patient of mine who was willing to come to this conference and exhibit 20 of his drawings and paintings. You will find some biographical information about him in the conference programme. The most important here, is that he was in psychiatric care being a concentration camp survivor and that as a routine, he had to do a Rorschach test. As a professional artist, he quickly understood what use he could make of this principle in order to expand his creative fantasy. I will show you three of his pictures: “The Squirrel”, “The Dead Blackbird”, and “The Kingfisher” (De Loos, 1995).

## Medical knowledge into the field of psychotraumatology

In 2004, Eric Vermetten wrote in Wolter's In Memoriam:

Wolter had a massive medical knowledge. Nevertheless, he also spoke with growing enthusiasm about developing his skill and experience in more reflective forms of psychological therapy. He had come to a more positive realization that his knowledge could be mobilized for better patient care by giving attention to the interpersonal context within which this information was shared with patients. This could be the operationalisation of his matrix diagnostics. In doing so, he also seemed to broaden his perspective to include considerations of past formative relationships and that much personal trauma related dysfunction could be contained and managed within particular personal and social contexts. In this, he seemed to acknowledge the limitations of reductionist explanations of post trauma reactions. Being ever the brave person he was, he talked with colleagues about very challenging patients. He is one of the very few that brought detailed medical knowledge in to the field of psychotraumatology. The field has benefited very much from his thoroughness; it has lost an inspiring, critical thinker.

## Continuing the ESTSS pathway

“As with his life, he was not done” (Vermetten, 2004), my father. In my own pathway in healthcare, I have seen how important it is for us to collaborate globally to improve quality and patient safety in healthcare for the most important member of the healthcare team: the victim, the victor and the perpetrator: our patients. When my father was seriously ill, he was still taking care of his own treatment until the final moment. As a home care nurse, also caring for him during his last few days, I advised him since the beginning of his illness to write down his medication list as I thought it could be unsafe for his treatment when entering a hospital without any information on the medication he was taking, and even prescribing himself. A few years later in my research in 2009 on the role of the patient in patient safety results show us that the only way to improve patient safety is to involve patients as partners of the healthcare team: the trauma stress care team (Van der Schrieck-De Loos, Posma & Salfischberger, 2009). Only patients participate during the entire care process. They can be a partner as they have a unique perspective on their care. Healthcare professionals can, while keeping full responsibility for their patients, improve their care by using the patient's eyes and ears. Therefore, patients can have an important role in preventing medical errors by providing information about how they take their medicine (Van den Bemt, Van der Schrieck-De Loos, Van der Linden, Theeuwes & Pol, accepted; Van der Schrieck-De Loos, 2013; Van der Schrieck-De Loos & Van Groenestijn, 2012, 2013). But, it depends on the patient's willingness and ability to become a partner in trauma stress care. Also, healthcare professionals need to invite their patients to partner with them to improve the patient's pathway and to make it safer to prevent, e.g., medication errors by interviewing patients on how they take their medicine as most important step in standardization of creating an accurate medication overview to reduce discrepancies (Van den Bemt et al., accepted; Van der Schrieck-De Loos, 2013; Van der Schrieck-De Loos & Van Groenestijn, 2012, 2013). In this context a good relationship with an active dialogue between healthcare professionals and patients is crucial and a starting point for implementation of the patient's role. Optimizing this relationship requires education of both patients and healthcare professionals by raising awareness and by using practical tools on patient involvement to improve patient safety and also care processes. To create a long-term effect of the patient's role, it is essential that incorporation of the patient's perspective is developed at the levels of the individual care process, healthcare organizations, the healthcare system and laws and regulations (Van der Schrieck-De Loos, 2013a).

## Patient as an ESTSS partner

To come back to combining the first president's pathway into ESTSS and my own pathway and vision on quality and patient safety improvement in healthcare, I see now that our paths intersect. Wolter ended his opening address 20 years ago by bringing one of his patients who was a great artist to the ESTSS Conference in Paris in 1995: patients as partners. Wolter's traumatized patients, the victim, the victor, and the perpetrator are still our patients who need to be involved in our traumatic stress care team to improve this important field of healthcare. Sharing and exchanging knowledge by involving patients at the micro-, macro-, meso-, and system level, even at conferences, could lead to the next step into the (clinical and patient safety) pathway of the ESTSS, which could be a suggestion for main objective number (8), contained with the ESTSS mission statement, and could be launched to celebrate the ESTSS 20th anniversary in Bologna as patients are not involved yet in the mission statement (European Society for Traumatic Stress Studies, 2013):

1. To increase and disseminate knowledge of traumatic stress based on good science.
2. To identify cross-European issues, such as differences in training and certification.
3. To stimulate cross-European training for different levels of certification.
4. To stimulate and help to set up local societies in different European countries.
5. To help establish European wide research on traumatic stress.
6. To focus on European issues relating to traumatic stress, e.g., disaster response, uniform services, child abuse, and so on.
7. To liaise with pan-European and international organizations/bodies.
8. To involve patients as partners of the traumatic stress care team into accelerating traumatic stress care across Europe and beyond.

In this way, I hope to contribute to the field of Wolter's work so that his footsteps into the also by his hand created ‘ESTSS pathway’ will continue with your patient, the victim, the victor, and the perpetrator, as an ESTSS partner to accelerate traumatic stress care.
